# Transcriptomic analysis at 48 h postmortem: a proof of concept for the identification of biomarkers to estimate time since death

**DOI:** 10.1007/s11033-025-11151-5

**Published:** 2025-10-21

**Authors:** Nahum Zepeta Flores, Luz María Garduño Zarazúa, Gabriela Piñón Zarate, Christian Adrian Cárdenas Monroy, Alejandra Mercado Salomon, Olivia Pérez Zamora, Carlos Pedraza Lara, Oliver Millán Catalán, Haydee Rosas Vargas, Silvia Jiménez Morales, Carlos Pérez Plasencia, Mariano Guardado Estrada

**Affiliations:** 1 Laboratorio de Genética, Escuela Nacional de Ciencias Forenses, Universidad Nacional Autónoma de México, Ciudad de México, México; 2 Unidad de Investigación Médica en Genética Humana, Unidad Médica de Alta Especialidad, Hospital de Pediatría, Centro Médico Nacional Siglo XXI, Instituto Mexicano del Seguro Social, Ciudad de México, Mexico; 3 Laboratorio de Inmunoterapia e Ingeniería de tejidos, Departamento de Biología Celular y Tisular, Facultad de Medicina, UNAM, Ciudad de México, México; 4 Laboratorio de Antropología y Odontología Forense, Escuela Nacional de Ciencias Forenses, Universidad Nacional Autónoma de México, Ciudad de México, México; 5 Laboratorio de Entomología Forense, Escuela Nacional de Ciencias Forenses, Universidad Nacional Autónoma de México, Ciudad de México, México; 6 Unidad de Investigación Biomédica en Cáncer, Instituto Nacional de Cancerología, Ciudad de México, Mexico; 7 Laboratorio de Innovación y Medicina de Precisión, Núcleo ″A″, Instituto Nacional de Medicina Genómica, Mexico, Mexico; 8 Laboratorio de Genómica, Unidad de Biomedicina, FES-IZTACALA, UNAM, Tlalnepantla, Mexico

**Keywords:** Transcriptome, Postmortem interval, MRNA, Microarray

## Abstract

**Background:**

The postmortem interval (PMI) refers to the time elapsed between an individual’s death and the examination of the body. Tissues undergo a sequence of anatomical changes following death, which are routinely used to estimate the PMI.

**Methods:**

To determine if these anatomical changes are associated with identifiable genomic adaptations that could characterize the PMI more accurately, we analyzed the rat skeletal muscle transcriptome at 0 and 48 h postmortem using Clariom™ S arrays. This study investigates whether specific transcriptomic changes correlate with PMI progression, offering a potential molecular tool to complement established anatomical methods.

**Results:**

A total of 3,873 differentially expressed mRNAs were identified, of which 2,787 downregulated and 1,086 upregulated transcripts. The most significantly downregulated mRNA was *Tnni1* (FC = -30.95, *p* = 1 × 10^−3^), while the most upregulated were *mt-ATP6*,* mt-ATP8*,* and mt-CO3* (FC > 7.78, *p* < 1.36 × 10^−12^). Gene ontology (GO) enrichment analyses revealed that mRNAs upregulated at 48 h in the PMI were primarily associated with vascular and endothelial processes, including nitric oxide transport and angiogenesis. Conversely, downregulated mRNAs were linked to mitochondrial activity and cellular metabolism, reflecting both a transient vascular response and metabolic pathway shutdown in the rat skeletal muscle.

**Conclusion:**

Our results demonstrate significant transcriptomic changes at 48 h postmortem, highlighting specific genes and biological pathways that may serve as candidate biomarkers for PMI estimation.

**Supplementary Information:**

The online version contains supplementary material available at 10.1007/s11033-025-11151-5.

## Introduction

The postmortem interval (PMI) is defined as the time elapsed between an individual’s death and the examination of the body, and is a critical parameter in forensic investigations [1]. During the early PMI (3–72 h after death), several morphological changes occur, including a drop in body temperature (*algor mortis*, AM), the onset of cadaveric rigidity (*rigor mortis*, RM), and the appearance of postmortem lividity (*livor mortis*, LM) due to blood pooling [1, 2]. Although these physical signs are traditionally used to estimate the time of death, their reliability is often compromised by external factors such as ambient temperature, humidity, and the individual’s physiological condition at the time of death [3, 4]. Consequently, identifying more precise molecular biomarkers for PMI estimation has become a priority.

This need is further underscored by the fact that as decomposition progresses, endogenous processes such as autolysis and putrefaction become dominant, and visual assessment becomes increasingly subjective and imprecise, especially beyond the early PMI window [5].

In 2007, Bauer underscored the significant potential of RNA-based methods to address key forensic challenges [6]. These include the identification of body fluids, estimation of wound age, assessment of biological stain age, elucidation of cause of death, and notably, the estimation of PMI [6]. Since then, extensive research has explored the use of RNA as a molecular marker in this context. RNA degradation has been widely investigated as a molecular marker for PMI estimation, particularly during the early stages (≤ 48 h). Most studies focus on a limited number of mRNAs and time points, often using RT-qPCR. While informative, this targeted approach constrains broader insights into postmortem molecular dynamics [7, 8]. As highlighted by Mroz et al. (2025), more comprehensive strategies are needed to improve accuracy in PMI estimation [9]. Transcriptome-wide analysis from 48 h PMI onward addresses this gap by offering a comprehensive overview of postmortem mRNA dynamics.

Modern molecular approaches have increasingly focused on the time-dependent degradation of biomarkers—particularly RNA—for more accurate PMI predictions. RNA is especially attractive due to its rapid degradation and its close temporal association with postmortem changes [10]. Unlike DNA and proteins, RNA is highly vulnerable to degradation by endogenous ribonucleases, bacterial activity, and environmental conditions, making it a promising candidate for PMI estimation. Advances in transcriptomics have further positioned RNA analysis as a pivotal area of forensic research [11]. Comprehensive transcriptome profiling studies have shown that RNA integrity is affected by various factors and that high-throughput sequencing technologies can be used to identify robust PMI biomarkers [12, 13].

It is well established that certain mRNAs can be detected at multiple postmortem intervals [14–19], and that changes in gene expression occur across species, including humans [20, 21]. These postmortem mRNA expression changes are thought to reflect biological processes involved in decomposition, such as apoptosis, autolysis, and immune responses [13, 22, 23]. Measuring degraded RNA transcripts can therefore provide insights into postmortem dynamics and support the development of mathematical models to improve PMI estimation [11].

Our research group has made important contributions to this field, including a comprehensive analysis of the miRNome in rat skeletal muscle during the early postmortem period. We identified a group of dysregulated miRNAs that strongly correlate with time since death [24]. We demonstrated that miR-381-3p and miR-23b-3p exhibit distinctive expression patterns in early PMI, supporting their potential as reliable biomarkers [25].

Building on these findings, the present study aims to further investigate molecular changes occurring after death. To gain a deeper understanding of transcriptomic alterations at 48 h postmortem, we will perform a global transcriptome analysis in rat skeletal muscle. This approach expands upon our previous work with miRNAs and will allow us to identify novel biomarkers for PMI estimation by examining gene expression changes on a genome-wide scale. Ultimately, this research seeks to contribute to the development of reliable molecular tools for accurate postmortem interval determination.

## Methods

### PMI rat model

A total of 10 adult male Wistar rats weighing less than 200 g were selected for this study, using a previously established PMI model in our laboratory [25]. The animals were divided into two groups: 0 h PMI (control, *n* = 5) and 48 h (*n* = 5) PMI. All animals were euthanized by cervical dislocation. The 48-hour PMI group was placed in a Binder KBW 240™ climatic chamber at a constant temperature of 25 °C for 48 h. After this period, both internal and external morphological changes were assessed in both groups.

A cephalocaudal physical examination was conducted to evaluate the presence or absence of typical early postmortem signs [26, 27], including AMLM, RM in the upper body (RMU), RM in the lower body (RML), drying (DR), generalized edema (ED), hair loss (HL), abdominal green discoloration (AGD), and abdominal distension (AD). Additionally, necropsy was performed to evaluate brain liquefaction (BL), brain edema (BE), liver discoloration (DL), loss of liver consistency (LLC), muscle livor mortis (MML), bowel swelling (BS), ascites (AS), and loss of muscle consistency (LMC).

Skeletal muscle samples (~ 200 mg) from the femoral region were collected and stored at − 80 °C until further analysis. Immediately after euthanasia, control group samples were similarly collected and stored. Both groups underwent complete postmortem physical examination.

### RNA extraction

Total RNA was extracted from skeletal muscle tissue using glass bead homogenization and Trizol™ Reagent. Briefly, 50–100 mg of frozen tissue was placed in a 2 mL tube containing 2 mm glass beads (ZR BashingBead™ Lysis Tubes, Zymo Research) and 1 mL of Trizol™. After homogenization, 200 µL of chloroform was added, the mixture vortexed, and centrifuged at 12,000 × g for 15 min at 4 °C. RNA was then purified according to the manufacturer’s protocol. RNA quantity was measured with a NanoDrop™ 2000 UV spectrophotometer (Thermo Scientific), and its integrity was qualitatively assessed by agarose gel electrophoresis following standard protocols [25]. RNA quality was confirmed in the control group (0 h) by the presence of distinct 28 S and 18 S rRNA bands. This assessment was omitted at 48 h of PMI due to significant expected degradation, which makes ribosomal banding an unreliable integrity indicator. For RNA yield and spectrophotometric absorbance ratios, see Table S5.

### Transcriptome analysis

Global gene expression profiling was performed using Clariom™ S arrays (Applied Biosystems). For hybridization, 10 ng of total RNA was amplified and biotin-labeled using the GeneChip™ 3′ IVT Pico Kit (Thermo Fisher Scientific). The resulting sense-strand cDNA targets were hybridized to Clariom™ S arrays at 45 °C for 16 h in a GeneChip™ Hybridization Oven 645 (Affymetrix). Arrays were then washed, stained, and scanned using the GeneChip™ Fluidics Station 450 and Scanner 3000 (Affymetrix).

Raw data were acquired with the GeneChip Command Console Software v4.0 and processed using the Robust Multi-array Average (RMA) algorithm in Transcriptome Analysis Console (TAC) Software v4.0.3.14. Differential expression analysis was performed using ANOVA with a threshold of fold change (FC) ≤ − 2 or ≥ 2 and *p* < 0.05. Data from this study are available in NCBI’s Gene Expression Omnibus under accession number GSE297166 (reviewer token: **ojgxyigwpzittqn**).

### Validation of mRNA expression by qRT-PCR

To validate microarray results, qRT-PCR was performed on independent samples in the same rats. Complementary DNA (cDNA) was synthesized from 500 ng of purified RNA using the High-Capacity cDNA Reverse Transcription Kit (Thermo Fisher Scientific).

PCR was first performed with GoTaq^®^ Green Master Mix (Promega) to determine optimal annealing temperatures (60 °C, 62 °C, and 64 °C), based on each primer’s Tm. Amplification products were resolved on 1.5% agarose gels.

Quantitative PCR was then conducted using SYBR™ Select Master Mix (Thermo Fisher Scientific) and gene-specific primers (see Table 1), on a LightCycler^®^ 480 II system (Roche). Cycling conditions were: 50 °C for 2 min (UDG activation), 95 °C for 2 min (polymerase activation), followed by 40 cycles of 95 °C for 15 s and 60 °C for 60 s. Relative mRNA levels were calculated using the ΔΔCt method, with GAPDH as the endogenous reference. GAPDH Ct values were stable across all samples.

**Table 1:** Sequences of primers for the amplification of selected genes evaluated by RT-PCR

Gene	Primer sequence (5′→3′)		Target size (bp)
	Sense	Antisense	
*Myh1*	AGTTGCATCCCTAAAGGCAG	TTCTGAGCCTCGATTCGCTC	142
*Mlf1*	TTTGGGAGGTTTGGCGGAAT	CCATTGAAAGCTGGCCAAAGT	121
*Adck3*	TTGGAA AGGTGCAGGGTCAG	CCCAGGGATGAAGACAGTGG	163
*Hbaa1*	AAGCAATCATGGTGCTCTCTG	GAAGACAATGAACAACCTCCC TA	119
*Ablim3*	CGAACTCGCTTAGAGCGCC	GGACTAGACTGTGTGTCAGGA	204
*Aqp1*	CACCTGCTGGCCATTGACTA	ATGCGGTCTGTAAAGTCGCT	188
*Gapdh* ^*a*^	GGGTGTGAACCACGAGAA ATA	CCCACTGTCGGTAAATGCTT	104

^a^Housekeeping gene transcript used as the normalization control for RT-PCR

### Biological processes of differentially expressed mRNAs

To identify biological processes associated with up- and downregulated genes, an Over-Representation Analysis (ORA) was conducted using WebGestalt (http://www.webgestalt.org/). Biological processes with a false discovery rate (FDR) ≤ 0.05 were considered significant.

### KEGG pathway enrichment analysis

Functional pathway enrichment was performed using DAVID (https://david.ncifcrf.gov/). Up- and downregulated mRNAs were submitted for KEGG pathway analysis. Pathways with *p* < 0.05 and FDR < 0.05 were considered significantly enriched.

### Ethics approval

All procedures were reviewed and approved by the local ethics and scientific committee, and by the Committee for the Care and Use of Laboratory Animals (CICUAL) of the Faculty of Medicine, National Autonomous University of Mexico (UNAM), under approval numbers 031–2024 and 005-CIC-2024. The study was conducted in strict accordance with national (NOM-062-ZOO-1999) and international guidelines for laboratory animal care and use.

## Results

### Morphological changes observed in rats at 48 h postmortem interval

The macroscopic changes observed at 48 of postmortem interval corresponded to the final phase of the early postmortem interval. In this stage the *livor mortis* or hypostasis was predominantly observed in the supine position on the plantar surfaces of the hind feet, specifically in areas devoid of hair (Figure S1, panels a and c). At this time, complete resolution of RM was observed in four rats and partial resolution in one (Figure S1, panel b). In the skin, loss of rigidity in the arrector pili muscles resulted in hair detachment with light friction over the abdominal surface (Figure S2, panels b and c).

Regarding putrefactive changes, a greenish discoloration of the abdominal wall was noted, especially pronounced in the right iliac fossa, where the cecum is located relatively close to the surface (Figure S2, panel a). As decomposition progressed, the greenish hue expanded throughout the abdomen, accompanied by AD due to gas accumulation produced by putrefactive bacteria, resulting in abdominal emphysema. This increase in intra-abdominal pressure led to distension of the intestinal loops in all specimens and caused protrusion of the eyeballs, tongue, and testes. A distinct greenish discoloration of the gonads was noted in four of the animals (Figure S3). At the cerebral level, a grayish coloration with partial loss of encephalic structure was observed, indicative of the liquefactive phase of decomposition. In the abdominal cavity, the liver exhibited heterogeneous dark brown discoloration, with no significant changes in consistency. These findings are consistent with previous reports in other mammalian models, including swine and human cadavers, where similar signs of livor mortis, abdominal distension, ocular collapse, and organ discoloration have been documented within the early PMI [2, 28].

### Transcriptomic analysis of rat skeletal muscle at 48 h of postmortem interval

**Fig. 1:**
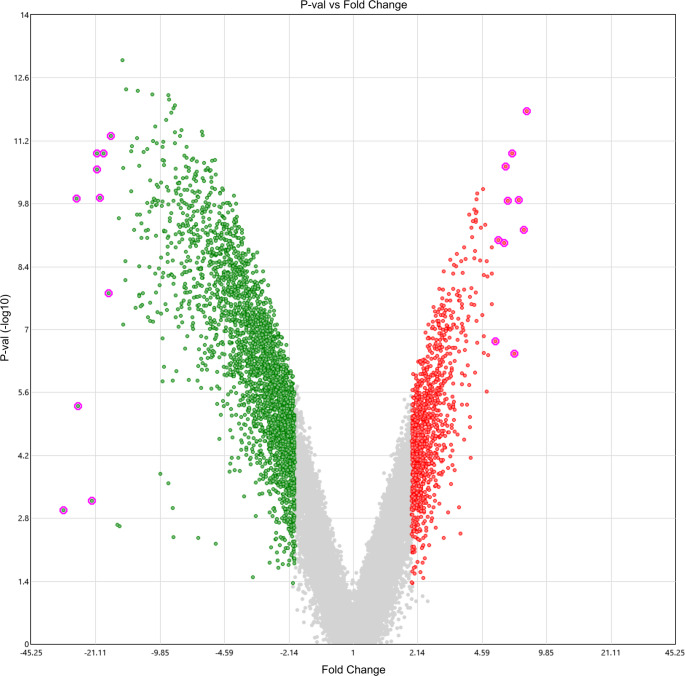
Volcano plot of mRNA expression at 48 h of PMI. The plot displays microarray results for 23,188 rat mRNAs, with −log10(p-value) plotted against log2(fold change). Using stringent thresholds (|fold change| ≥ 2 and *p* ≤ 0.05), we identified 3,873 differentially expressed genes (16.7% of total). Among these, 2,787 genes (71.96%) were downregulated (green) and 1,086 (28.04%) were upregulated (red). The most statistically significant upregulated and downregulated mRNAs are highlighted (see Methods for selection criteria)

The rat skeletal muscle transcriptome was analyzed at 48 h postmortem using the Clariom™ S array (Applied Biosystems). Expression profiles were compared with those of the control group (0 h PMI) to identify differentially expressed genes. Out of the 23,188 genes explored in the microarray, 3,873 (16.7%) met the established criteria for differential expression (Fold Change ≤ −2 or ≥ 2, and p-value ≤ 0.05). Among these, 1,086 genes (28.04%) were upregulated, while 2,787 genes (71.96%) were downregulated at 48 h *postmortem* (Fig. 1; Supplementary Tables S1 and S2).

The ten most significantly upregulated and downregulated mRNAs are listed in Table 2. Respect to the downregulated genes, we found genes associated with muscle function as Tnnt1, Adck3, Myh2, and Myh7. Interestingly, the most upregulated genes corresponded mainly to the mitochondrial genes as *MT-ATP6*,* MT-ATP8*, and *MT-COX3*. Moreover, there was also upregulation of *Hba-a1* and *Hba-b1* genes. It is important to highlight that the fold-change difference between the most downregulated gene and the most upregulated gene was 5.7-fold.

**Table 2:** Top ten most Up- and Down-Regulated mRNAs after 48 h of the Post-Mortem interval

Gene Symbol	Description	Fold change (FC)	Affymetrix probe set ID	*p*-value	FDR *p*-value
*Downregulated*					
Tnnt1	Troponin T type 1 (skeletal, slow)	−30.95	TC0100000869.rn.2	1 × 10^−03^	4.2 × 10^−03^
Adck3	aarF domain containing kinase 3	−26.56	TC1300002059.rn.2	1.20 × 10^−10^	2.53 × 10^−08^
Myh2	Myosin, heavy chain 2, skeletal muscle, adult	−26.16	TC1000000862.rn.2	4.96 × 10^−06^	5.22 × 10^−05^
Myh7	Myosin, heavy chain 7, cardiac muscle, beta	−22.07	TC1500001690.rn.2	6 × 10^−04^	2.8 × 10^−03^
Mlf1	Myeloid leukemia factor 1	−20.9	TC0200001344.rn.2	2.68 × 10^−11^	1.19 × 10^−08^
Actn2	Actinin alpha 2	−20.83	TC1700002039.rn.2	1.19 × 10^−11^	8.79 × 10^−09^
Myot	Myotilin	−20.13	TSUnmapped00000070.rn.2	1.17 × 10^−10^	2.51 × 10^−08^
Myh1	Myosin, heavy chain 1, skeletal muscle, adult	−19.2	TC1000004347.rn.2	1.20 × 10^−11^	8.79 × 10^−09^
Csrp3	Cysteine and glycine-rich protein 3 (cardiac LIM protein)	−18.15	TC0100005464.rn.2	1.54 × 10^−08^	5.46 × 10^−07^
Myom1	Myomesin 1	−17.64	TC0900001342.rn.2	4.90 × 10^−12^	6.41 × 10^−09^
*Upregulated*					
ATP6; ATP8; COX3	ATPase subunit 6; ATPase subunit 8; cytochrome c oxidase subunit 3	7.78	TC0700002260.rn.2	1.36 × 10^−12^	3.46 × 10^−09^
Hba-a1	Hemoglobin alpha, adult chain 1	7.51	TSUnmapped00000112.rn.2	5.96 × 10^−10^	6.03 × 10^−08^
Hbb-b1;	Hemoglobin, beta adult major chain	7.06	TCUn_AABR07024041v100000001.rn.2	1.29 × 10^−10^	2.63 × 10^−08^
LOC289647	SUMO specific peptidase 2, pseudogene 6	6.71	TC1400001719.rn.2	3.42 × 10^−07^	6.04 × 10^−06^
LOC102554992	Similar to 60 S ribosomal protein L29	6.58	TSUnmapped00000058.rn.2	1.21 × 10^−11^	8.79 × 10^−09^
LOC689064	Beta-globin	6.2	TC0700003996.rn.2	1.36 × 10^−10^	2.69 × 10^−08^
Gabra4	Gamma-aminobutyric acid type A receptor subunit alpha4	6.05	TC1400000593.rn.2	2.36 × 10^−11^	1.19 × 10^−08^
LOC689064	Hemoglobin, beta adult s chain	5.96	TCUn_AABR07024041v100000002.rn.2	1.20 × 10^−09^	9.18 × 10^−08^
Hbb-b1	hemoglobin, beta adult major chain	5.58	TC1000004399.rn.2	1.02 × 10^−09^	8.29 × 10^−08^
Zfp101	Zinc finger protein 101	7.78	TC0M00000015.rn.2	1.82 × 10^−07^	3.60 × 10^−06^

**Fig. 2:**
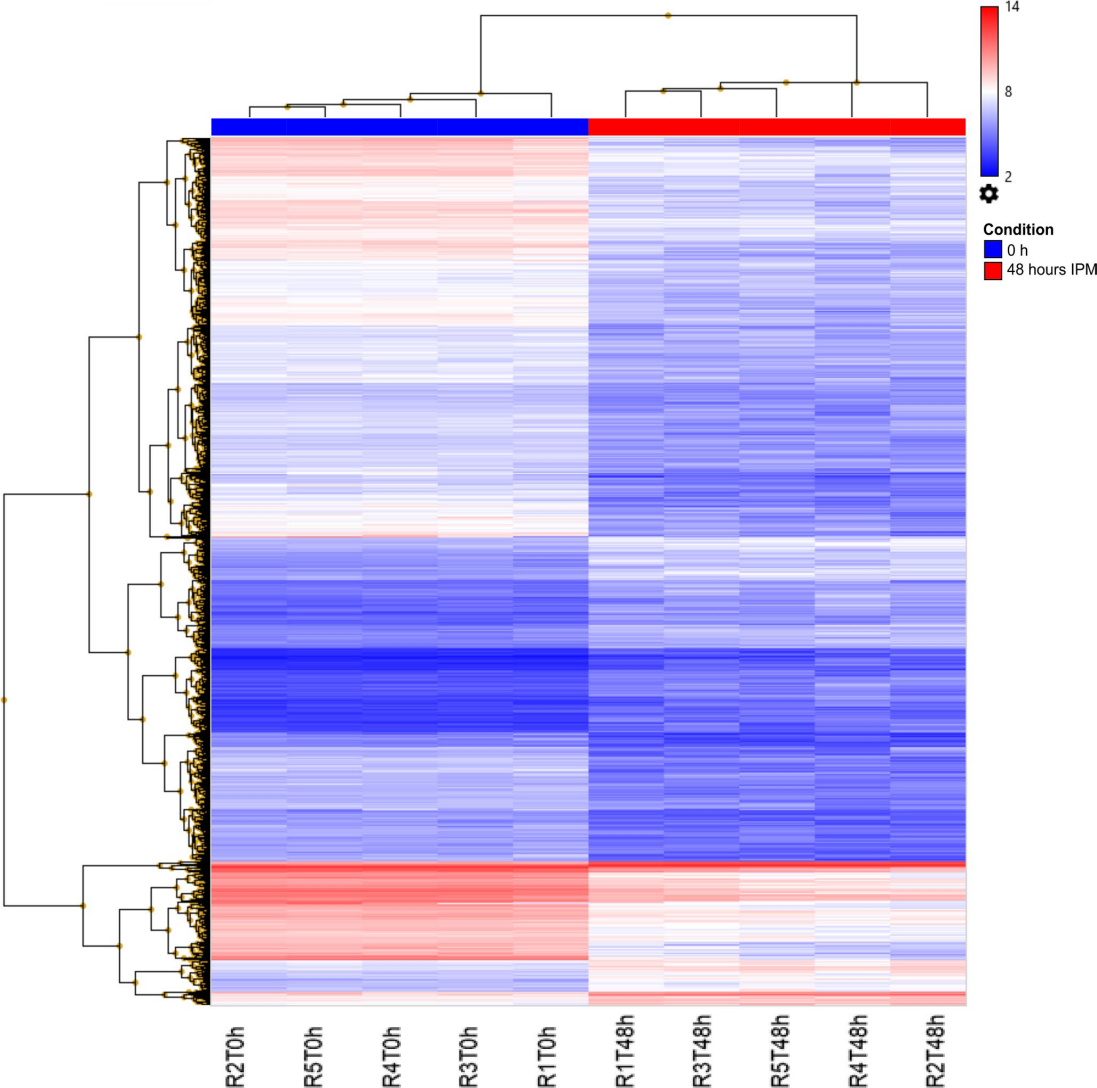
Hierarchical clustering dendrogram of 3873 differentially expressed mRNAs at 48 h of PMI. The dendrogram displays expression patterns of 3,873 significantly altered mRNAs (Fold change ≥ or ≤ 2, *p* ≤ 0.05) compared to control samples. Gene expression levels are color-coded, with red indicating upregulated mRNAs and blue representing downregulated mRNAs. Color intensity corresponds to the magnitude of fold change (darker hues represent more pronounced expression changes)

Hierarchical clustering analysis revealed at least four distinct gene clusters exhibiting significant shifts in expression. These clusters represented groups of genes transitioning from upregulated to downregulated states or vice versa, reflecting dynamic regulatory changes occurring during the *postmortem* interval. Importantly, the expression patterns of the most significantly altered mRNAs distinctly separated the 48-hour PMI group from the control group, as shown in the dendrogram (Fig. 2). One cluster showed a pronounced upregulation at 48 h *postmortem*, while the remaining three were predominantly downregulated compared to controls. Collectively, these findings demonstrate that substantial transcriptomic alterations occur in rat skeletal muscle by 48 h postmortem, supporting the potential use of mRNA expression profiles as reliable molecular biomarkers for PMI estimation.

### Validation of microarray data by RT-qPCR

RT-qPCR was employed to validate microarray findings and assess differential expression estimates, as this method is widely recognized for confirming transcriptomic results identifying potential biases inherent to microarray-based quantification [29]. In line with standard validation practices, we selected protein-coding genes for RT-qPCR analysis among the most strongly dysregulated transcripts, prioritizing those with substantial fold changes and robust expression levels to ensure reliable amplification [30].

To validate the gene expression changes observed in the microarray analysis, six mRNAs were selected for confirmation using real-time quantitative PCR (RT-qPCR). The upregulated genes included *Hba-a1*,* Aqp1*, and *Ablim3*. Although Aqp1 and Ablim3 were not among the top-ten most significant genes, they ranked within the top 50 (see Supplemental Table S1). On the other hand, the downregulated genes *Adck3*,* Mlf1*, and *Myh1* were selected for RT-qPCR validation.

The RT-qPCR results were consistent with the microarray findings, confirming the direction and significance of gene expression changes at 48 h postmortem. Among the upregulated genes, *Hba-a1* showed the highest fold change, with a fold change (FC) of 7.51 in the microarray and 114.28 in the RT-qPCR validation (*p =* 0.0079). Interestingly, while microarray analysis showed moderate upregulation of *Aqp1* (4.69-fold) and *Ablim3* (4.63-fold), RT-qPCR revealed substantially greater upregulation (24.40-fold for *Aqp1* and 47.62-fold for *Ablim3*; *p* = 0.0079 for both).

**Fig. 3:**
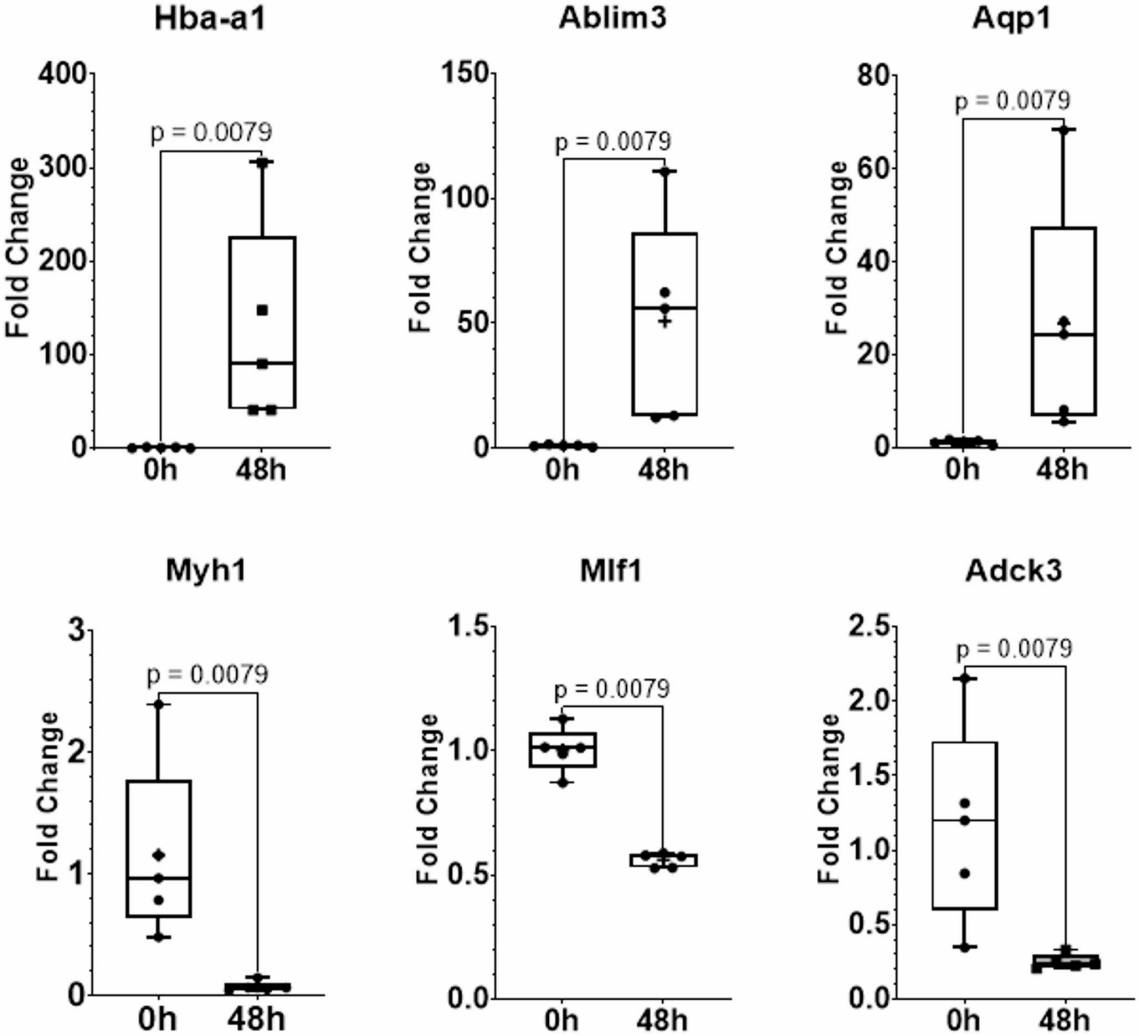
Validation of microarray expression data using RT-PCR. Based on microarray expression profiling, we selected three up-regulated genes (*Hba-a1*,* Ablim3*, and *Aqp1*) and three down-regulated genes (*Myh1*,* Mlf1*, and *Adck3*) for RT-qPCR validation. Gene-specific primers were designed and used to quantify transcript levels. Differential expression between 0-hour (control) and 48-hour postmortem intervals was assessed using the non-parametric Mann-Whitney U test (*p* < 0.05)

Downregulated genes showed consistent expression patterns between both platforms. RT-qPCR analysis confirmed significant downregulation of *Adck3* (4.65-fold decrease), *Mlf1* (1.79-fold decrease), and *Myh1* (15.57-fold decrease) (all *p* = 0.0079). While the absolute fold-change values differed between microarray and RT-qPCR analyses, the direction of regulation was concordant. These findings validate our microarray results and demonstrate significant transcriptomic remodeling in rat skeletal muscle at 48 h postmortem (Fig. 3).

### Gene ontology enrichment analysis of biological processes for upregulated mRNAs at 48 h PMI

**Fig. 4:**
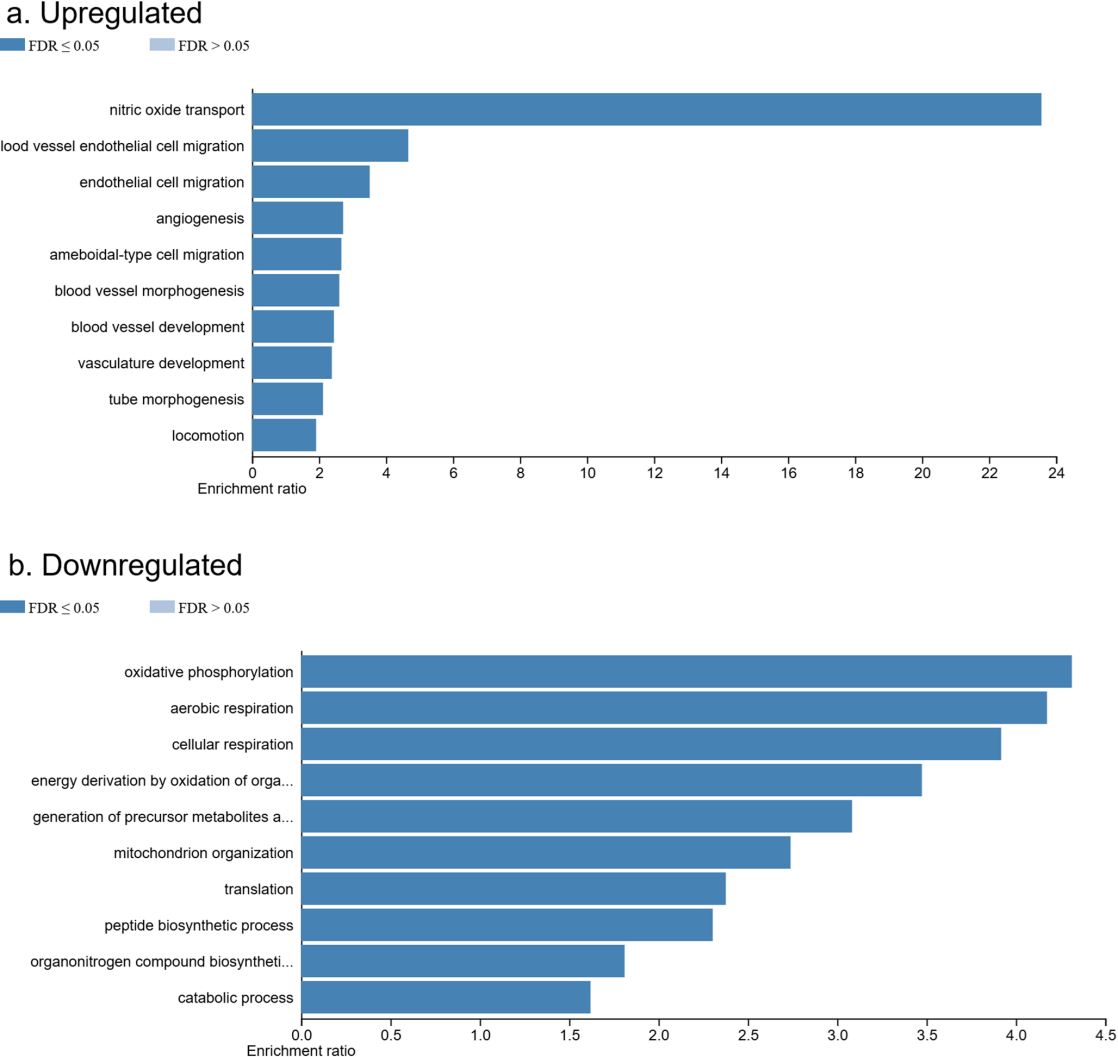
Gene Ontology Enrichment Analysis of Target Genes. This figure displays the primary biological pathways associated with the target genes of the upregulated (**a**) and downregulated (**b**) mRNAs at 48 h of PMI. The x-axis represents the enrichment ratio, indicating the strength of pathway association for each gene set

An over-representation analysis of GO biological processes was conducted using the 1,086 upregulated mRNAs identified in rat skeletal muscle at 48 h postmortem. Of the 854 submitted user IDs, 834 were successfully mapped to unique Entrez Gene IDs, while the remaining 20 could not be mapped and were excluded from the analysis. From the mapped IDs, 513 were annotated to the selected GO functional categories and included in the final enrichment analysis. The results, shown in Fig. 4 (panel a), revealed that the most significantly enriched biological processes (FDR ≤ 0.05) were primarily associated with vascular and endothelial functions. These included nitric oxide transport (GO:0030185), blood vessel endothelial cell migration (GO:0043534), endothelial cell migration (GO:0043542), angiogenesis (GO:0001525), blood vessel morphogenesis (GO:0048514), blood vessel development (GO:0001568), vasculature development (GO:0001944), and tube morphogenesis (GO:0035239). Additionally, terms such as ameboidal-type cell migration (GO:0001667) and locomotion (GO:0040011) were also significantly enriched, indicating the activation of cellular motility and vascular remodeling pathways during the early postmortem interval. These findings suggest that, despite the overall metabolic shutdown after death, there is a transient upregulation of genes related to vascular dynamics and endothelial responses in skeletal muscle. This may reflect a final adaptive or compensatory cellular reaction—perhaps an attempt at reorganization—prior to the irreversible collapse of function during the postmortem period.

### Gene ontology enrichment analysis of biological processes for downregulated mRNAs at 48 h PMI

On the other hand, an over-representation analysis of GO biological processes was performed using the 2,787 downregulated mRNAs identified in rat skeletal muscle at 48 h postmortem. From the initial dataset of 2,472 user IDs, 2,445 were successfully mapped to unique Entrez Gene IDs, while 27 could not be mapped and were excluded from further analysis. Of the mapped genes, 1,655 were annotated to the selected GO functional categories and included in the final enrichment analysis. The results, shown in Fig. 4 (panel b), highlight the most significantly enriched biological processes affected by the downregulated mRNAs (FDR ≤ 0.05). These processes were primarily related to mitochondrial function and cellular metabolism. Among the most significantly impacted were oxidative phosphorylation (GO:0006119), aerobic respiration (GO:0009060), cellular respiration (GO:0045333), and energy derivation by oxidation of organic compounds (GO:0015980), all of which are fundamental for cellular energy production. Additionally, processes such as the generation of precursor metabolites and energy (GO:0006091) and mitochondrion organization (GO:0007005) were enriched, underscoring the disruption of mitochondrial structure and function. Other affected biological processes included translation (GO:0006412), peptide biosynthetic processes (GO:0043043), and organonitrogen compound biosynthetic process (GO:1901566), indicating a broad suppression of protein synthesis and anabolic activity. Interestingly, the enrichment of the catabolic process (GO:0009056) term also suggests a decline in substrate degradation pathways. Collectively, these findings reveal that the postmortem interval leads to a marked shutdown of transcriptional activity associated with energy metabolism, protein synthesis, and mitochondrial organization in skeletal muscle tissue following death.

### KEGG pathway analysis of upregulated mRNAs at 48 h of PMI

A total of 1,086 upregulated mRNAs identified at 48 h postmortem interval (PMI) were submitted to KEGG pathway enrichment analysis using the DAVID database, with *Rattus norvegicus* as the background reference. Of these, 830 mRNAs were successfully mapped to DAVID IDs. The analysis initially identified 50 KEGG pathways as impacted, of which 36 showed statistical significance (*p* < 0.05). After applying a false discovery rate (FDR) correction (FDR ≤ 0.05), 10 pathways remained significantly enriched. These included: olfactory transduction (rno04740), African trypanosomiasis (rno05143), renin secretion (rno04924), axon guidance (rno04360), cGMP–PKG signaling pathway (rno04022), vascular smooth muscle contraction (rno04270), platelet activation (rno04611), retrograde endocannabinoid signaling (rno04723), oxytocin signaling pathway (rno04921), and aldosterone synthesis and secretion (rno04925) (Supplementary Table S3). These findings suggest that, beyond structural and metabolic alterations, the postmortem transcriptomic profile also reflects activation of pathways involved in signaling, vascular response, and neuroendocrine regulation.

### KEGG pathway analysis of downregulated mRNAs at 48 h of PMI

A total of 2,787 downregulated mRNAs identified at 48 h postmortem interval (PMI) were analyzed for KEGG pathway enrichment using the DAVID database, with Rattus norvegicus as the background. Of these, 2,443 mRNAs were successfully mapped to DAVID IDs. The analysis initially identified 106 KEGG pathways as impacted, of which 87 were statistically significant (*p* < 0.05). After applying a FDR correction (FDR ≤ 0.05), 71 pathways remained significantly enriched. These included several neurodegenerative disease pathways such as Parkinson disease (rno05012), Huntington disease (rno05016), prion disease (rno05020), and Alzheimer disease (rno05010), as well as broader neurodegeneration-related pathways like pathways of neurodegeneration—multiple diseases (rno05022). Other significantly enriched pathways included thermogenesis (rno04714), amyotrophic lateral sclerosis (rno05014), diabetic cardiomyopathy (rno05415), oxidative phosphorylation (rno00190), and non-alcoholic fatty liver disease (rno04932), among others (Supplementary Table S4). These results highlight the widespread downregulation of genes involved in mitochondrial function, energy metabolism, and neuronal integrity, reflecting systemic transcriptomic decline and cellular dysfunction at 48 h postmortem.

## Discussion

Using microarray data, we analyzed the transcriptome at 48 h PMI. We found that 16.7% (*n* = 3,873) of the total analyzed mRNA transcripts showed altered gene expression at 48 h PMI meeting the significance threshold (fold change ≤ − 2 or ≥ 2 and *p* < 0.05). Among these, 1,086 were upregulated and 2,787 were downregulated in skeletal muscle. This means that at least 2.5 times more were downregulated than upregulated at 48 h of time since death. Among the upregulated genes, we found the mitochondrial genes *MT-ATP6*,* MT-ATP8*,* MTCOX3*, while the most downregulated genes were *Tnnt1*,* Adck3*, and *Myh2*. We also validated the expression of some of these genes using RT-qPCR. When exploring the functions and pathways of the altered genes, we found that the upregulated ones mainly were associated with vascular dynamics, such as angiogenesis, while the downregulated genes participated in metabolic processes like oxidative phosphorylation.

The morphological postmortem changes observed in rats including livor mortis, abdominal distension, ocular collapse, and organ discoloration, were consistent with those in humans, particularly *in LM*,* RM*, and putrefaction [26]. Comparable findings have also been reported in human cadavers [2] and swine models [28]. These parallels indicate that the rat model is suitable for identifying biomarkers to estimate the PMI accurately and reinforce its applicability for forensic comparisons. Following the morphological evaluation, which demonstrated the usefulness of the rat model for PMI biomarker research, microarray analysis was conducted. The expression of six mRNAs—three downregulated (*Adck3*,* Mlf1*,* Myh1*) and three upregulated (*Hba-a1*,* Aqp1*,* Ablim3*)—was validated by RT-qPCR. The RT-qPCR results confirmed the microarray data, showing consistent and statistically significant expression trends, although fold change values differed between the two techniques. This consistency is important for further exploration of these genes through different postmortem intervals.

The proportion of downregulated genes is higher than the proportion of upregulated genes, which could be related with degradation of mRNA through the PMI. Moreover, this downregulation could be related by the autolysis process itself since the most downregulated genes participate in processes related with muscle contraction and motor function that could be due to *RM* [27]. Despite the autolytic process, certain housekeeping genes such as GAPDH remain relatively stable during the early postmortem period, making them suitable internal controls for gene expression quantification by RT-qPCR, as previously reported in postmortem human tissue studies [31]. Interestingly, the loss of function of TNNT1 in a mouse model produced a myopathy in animals carrying mutation in this gene [32]. Also, *Myh1*, have been reported in muscle age degenerative processes [33].

In contrast, the upregulated mitochondrial genes *MT-ATP6*,* MT-ATP8*, and *MT-COX3* indicated sustained metabolic activity, consistent with the known postmortem persistence of mitochondrial integrity [34]. Interestingly, we found also an upregulation of genes related the structure of hemoglobin as the heavy chains (Hba-a1 and Hbb-b1). These are structural genes which can be used as housekeeping genes to normalize the gene expression of other genes. However, their over expression in PMI could be related with lower tissue concentrations of oxygen. For instance, it has been reported and upregulation of HBA1 in humans in postmortem tissues [21].

Hierarchical clustering revealed distinct mRNA expression patterns between control and 48-hour PMI groups, with four major clusters showing dynamic regulation: three shifting from up- to downregulation and one in the opposite direction. This supports the existence of temporally regulated gene expression after death, consistent with findings in human brain tissue [23]. Moreover, gene ontology over-representation analysis of the upregulated mRNAs revealed enrichment in biological processes related to angiogenesis, endothelial cell migration, vascular morphogenesis, and nitric oxide transport, suggesting activation of vascular remodeling and stress adaptation responses after death [35–41].

KEGG pathway enrichment analysis of upregulated genes revealed 10 statistically significant pathways (FDR ≤ 0.05), including vascular smooth muscle contraction, platelet activation, renin secretion, and aldosterone signaling. While some enriched pathways (e.g., olfactory transduction, African trypanosomiasis) may be artifacts, others reflect regulatory systems that could be activated in postmortem [40].

The convergence of GO and KEGG analyses underscores the biological coherence of vascular remodeling, suggesting an attempt to maintain perfusion and initiate local responses to tissue damage [42–44]. Conversely, the GO analysis of the 2,787 downregulated mRNAs revealed suppression of processes related to oxidative phosphorylation, aerobic respiration, mitochondrial organization, translation, and biosynthetic activities, which are hallmarks of metabolic shutdown after death [45–48].

KEGG analysis of the downregulated genes highlighted pathways associated with mitochondrial dysfunction and neurodegenerative diseases (e.g., Parkinson’s, Alzheimer’s, Huntington’s, ALS), consistent with oxidative stress and impaired energy metabolism [45–49]. These findings provide a comprehensive view of the molecular changes occurring in skeletal muscle 48 h after death and support the use of transcriptomic signatures as potential biomarkers for estimating the postmortem interval.

This study has several important limitations to acknowledge. As a preliminary investigation, our sample sizes were limited. However, we applied stringent statistical criteria to identify the most significant genes, and were able to validate several candidates (including Aqp1 and Ablim1) that did not rank among the top significant mRNAs. Future studies with larger sample sizes and human translation will be necessary to further explore these findings. It should be noted that while our study employed controlled postmortem conditions, these may not fully replicate the degradation processes occurring in actual forensic scenarios. Postmortem degradation patterns can vary significantly depending on both the cause of death and premortem conditions, which may affect the generalizability of our results.

Our findings demonstrate that at this postmortem interval (48 h), there is marked upregulation of genes involved in physiological processes such as angiogenesis and metabolism, along with stress-response pathways associated with hypoxic conditions. We also observed activation of muscle degradation pathways. A better understanding of these altered metabolic pathways could facilitate the identification of potential biomarkers for postmortem interval estimation.

## Conclusions

Transcriptional profiling of rat skeletal muscle at 48 h postmortem revealed a complex pattern of gene regulation, with mitochondrial genes *Tnni1*,* MT-Atp6*, *MT-Atp8*, and *MT-Cox3*) emerging as particularly promising biomarkers for postmortem interval (PMI) estimation. Our findings demonstrate that the rat model provides biologically relevant data that may inform human PMI estimation and contribute to human identification and cause-of-death determination. These results offer important insights into the molecular mechanisms underlying early postmortem changes and establish a framework for developing RNA-based PMI estimation tools. However, additional research, including comprehensive time-course analyses, validation in human tissues, and investigation of how premortem conditions affect these markers, will be essential to establish their forensic utility.

## Supplementary Information

Below is the link to the electronic supplementary material.

Supplementary Material 1

Supplementary Material 2

Supplementary Material 3

Supplementary Material 4

Supplementary Material 5

Supplementary Material 6

Supplementary Material 7

Supplementary Material 8

## Data Availability

Data from this study are available in NCBI’s Gene Expression Omnibus under accession number [GSE297166](https:/www.ncbi.nlm.nih.gov/geo/query/acc.cgi? acc=GSE297166) (reviewer token: ojgxyigwpzittqn).
