# Supplementary figures and images for: Transcriptomic analysis at 48 h postmortem: a proof of concept for the identification of biomarkers to estimate time since death
Source: Mol Biol Rep. 2025 Oct 21;52(1):1050. doi: 10.1007/s11033-025-11151-5 (PMC12540568; doi:10.1007/s11033-025-11151-5)

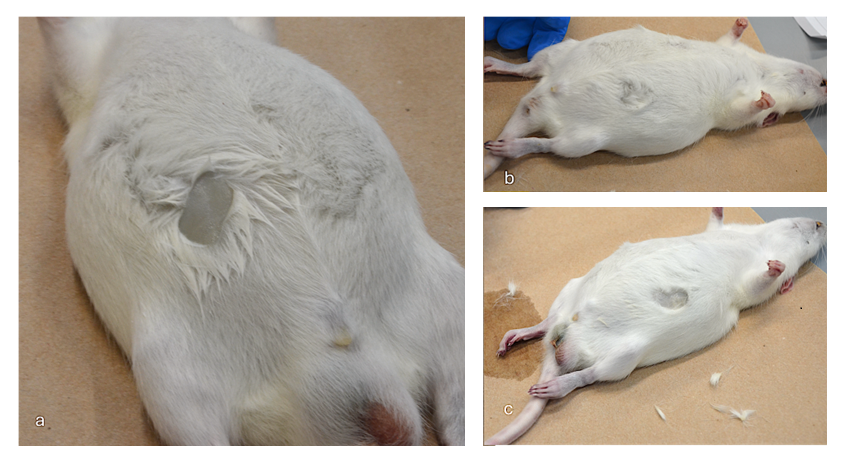

Supplement: Supplementary file 1 — Supplementary Material 1 [file 11033_2025_11151_MOESM1_ESM.png]

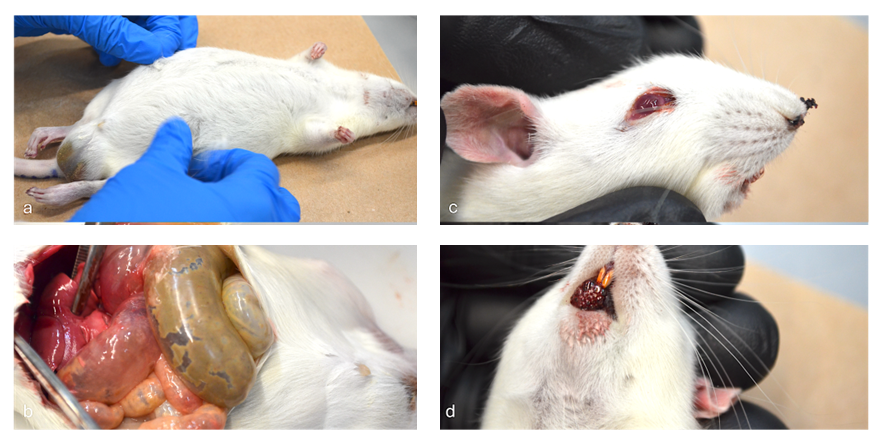

Supplement: Supplementary file 2 — Supplementary Material 2 [file 11033_2025_11151_MOESM2_ESM.png]

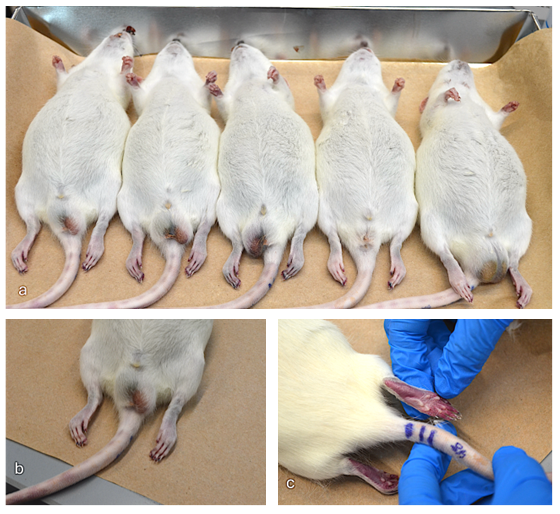

Supplement: Supplementary file 3 — Supplementary Material 3 [file 11033_2025_11151_MOESM3_ESM.png]
